# Dantrolene in the treatment of malignant hyperthermia: a case report

**DOI:** 10.1186/1471-2253-14-S1-A7

**Published:** 2014-08-18

**Authors:** Gunilla Islander

**Affiliations:** 1 Department of Anesthesia, Lund University Hospital, Lund, 22185 Sweden

## Background

Malignant hyperthermia (MH) is a rare disorder, occurring in 1 per 5,000 to 1 per 50,000 – 100,000 anaesthetic procedures and is usually fatal if untreated. Relapse occurs in 23% of patients in spite of appropriate management. Since emergency treatment is required, few reports document the time course of events relative to treatment.

## Case report

The patient, a 19 year old healthy male, who suffered childhood asthma, was anaesthetised for a planned mandible sagittal split and bimaxillar osteotomia due to prognathia. Anaesthesia was induced with propofol 140mg and suxamethonium 100mg was administered for muscle relaxation. Relaxation was not optimal but intubation was performed without problems. Anaesthesia was maintained with remifentanil (0.6-2.3µg/kg/min) and desflurane (4.9-7vol%). After 15 minutes the airway pressure was 30mbar, which was initially interpreted as broncho-obstructivity due to his childhood asthma, however, it was probably a sign of muscle rigidity. The patient was given minute ventilation at 8.2 litres, with pCO_2_ subsequently remaining stable for 2 hours. However, this increased to 10kPa and the patient began to sweat. The patient’s temperature rose from 35.5°C 15 minutes after the start of the procedure to 37.9°C after 2.5 hours, but 15 minutes later the patient’s temperature was 38.9°C. MH was diagnosed and dantrolene 380mg administered. Minute ventilation was increased to 12 litres FiO_2_ 100%. Cooling was performed with cold intravenous Ringer Acetate and surface cooling with ice. After 25 minutes the temperature was 37.6°C. The patient remained on the ventilator for 11 hours after anaesthesia. There was mild recrudescence at approximately 6 hours, with blood pH gradually falling from 7.43 to 7.31, and pCO2 increasing from 4.9 to 6.8. A 200mg dantrolene infusion was therefore administered over 2 hours, prior to extubation of the patient, such that the total dose of dantrolene administered was 580mg (29 vials). When the patient came round he reported severe muscle pain. With regard to surgery, the mandible sagittal split was performed, but the maxillary surgery was cancelled, leaving the patient unable to close his mouth or bite. Over the following 2 weeks, prior to the second surgery, the patient was only able to drink and lost 6kg. Second surgery was performed in trigger free anaesthesia without problems. In vitro contracture test revealed MH susceptible results (halothane contracture 2.8g and caffeine 1.3g). The patient has p. 552 Arg>Trp mutation in RYR1.

## Conclusions

This report describes the time course for the onset of clinical features of MH, and associated changes in laboratory parameters, and during relapse. It clearly demonstrates the need for, and efficacy of, dantrolene, when used immediately. Since the introduction of intravenous dantrolene, mortality from MH has fallen from 80% to less than 5%. The European Guidelines for the management of a MH crisis recommend the immediate use of dantrolene sodium in combination with other symptomatic treatments as soon as a MH crisis is suspected, and recognise that an adult patient requires up to 50 vials for effective treatment.

